# Humanities in medicine: preparing for practice

**DOI:** 10.1007/s40037-013-0086-8

**Published:** 2013-10-04

**Authors:** Daryl Ramai, Shoshanna Goldin

**Affiliations:** 1 School of Medicine, St. George’s University, True Blue, Grenada, West Indies; 2 Department of Interdisciplinary Studies - Global Health, Wake Forest University, Winston-Salem, NC 27109 USA

As medical curriculum continues to experience a renaissance into the twenty first century, there comes to birth a variety of seeded programmes in which there is no concrete definition for what is termed humanities in medicine. However, while the humanities continues to play an integral role in medicine, alongside the publication of numerous articles published by medical journals, medical institutions have tailored related programmes intended to support students’ learning about the social and cultural contexts of health, illness, and medical care. Others have defined a broader paradigm to include history, philosophy, literature, and arts. Though there is much versatility in these programmes, the expected outcomes have been collectively agreed upon. This article highlights three primary domains for why humanities should be incorporated into medical circular and the training of ‘tomorrow’s doctors’.

Firstly, according to the Association for Medical Humanities, the study of humanities contributes to the development of students’ and practitioners’ capacity to listen, interpret, and communicate, while fostering an appreciation for the ethical dimensions of practice [1]. This places more focus on the patient as a whole, assessing both objective and subjective experiences of illness and health. For example, by understanding the subjective nature of an individual’s behavioural patterns, a doctor could find it beneficial to under-prescribe. The study of humanities may assist in nurturing students in becoming more insightful, reflective, and hopefully more influential in shaping the course of health care. This degree of introspective thinking facilitates and encourages a willingness and capacity for innovation surrounding larger public debates such as with the Universal Health Care Act within the United States [2].

Secondly, humanities in medical education can foster an active professional conscience in students. Faunce [3] has suggested that this component of student education begins by encouraging the practical expression of foundational virtues such as empathy, compassion, fairness, and loyalty to the relief of patients’ suffering. With the rigors of medical study and practice, students and doctors sometimes lose sight of the patient, much less for these essential principles. It has been well documented that students lose empathy during their medical education. By incorporating and re-enforcing these values earlier and throughout medical school, students will eventually adapt and grow into these ideals as part of their own identity, making it an automatic and habitual response.

Thirdly, by creating a relationship between humanities and medicine, student doctors and physicians are provided with an outlet to express themselves in a safe and responsible manner. There is also evidence that suggests that humanities may aid in the health of students and practitioners. While practitioners are entrusted with the lives of others, they share in the burden of witnessing the toll of disease. The intensity of the constant struggle to provide care and fight potential loss can build barriers between patients and physicians. By incorporating humanities into medicine, we allow physicians to direct and express their fears, stress, and hopes in a secure arena. With such potential, medical schools are growing increasingly aware of the importance of incorporating humanities into the curriculum. In 2011, a medical humanities course was required for graduation at 52 % of American medical schools [4].

In conclusion, the value of humanities in the medical profession is multi-faceted. From the classrooms of medical school to the operating rooms of the hospital, tomorrow’s doctors will be responsible for absorbing and providing medical knowledge with attention and care. As the medical profession continues to include humanities in its curriculum, it will have an opportunity to develop practical and valuable skills such as critical and reflective thinking, a professional conscience, and a healthy emotional outlet for the next generations of physicians.
